# Multiscale Modeling of Bistability in the Yeast Polarity Circuit

**DOI:** 10.3390/cells13161358

**Published:** 2024-08-15

**Authors:** Siarhei Hladyshau, Kaiyun Guan, Nivedita Nivedita, Beverly Errede, Denis Tsygankov, Timothy C. Elston

**Affiliations:** 1 School of Biological Sciences, Georgia Institute of Technology, Atlanta, GA 30332, USA; 2 Wallace H. Coulter Department of Biomedical Engineering, Georgia Institute of Technology and Emory University, Atlanta, GA 30332, USA; 3 Curriculum in Bioinformatics and Computational Biology, University of North Carolina at Chapel Hill, Chapel Hill, NC 27599, USA; 4 Department of Pharmacology, University of North Carolina at Chapel Hill, Chapel Hill, NC 27599, USA; 5 Department of Biochemistry and Biophysics, University of North Carolina at Chapel Hill, Chapel Hill, NC 27599, USA; 6 Computational Medicine Program, University of North Carolina at Chapel Hill, Chapel Hill, NC 27599, USA

**Keywords:** biochemical networks, Cdc42 Rho GTPase, Turing instability, hysteresis

## Abstract

Cell polarity refers to the asymmetric distribution of proteins and other molecules along a specified axis within a cell. Polarity establishment is the first step in many cellular processes. For example, directed growth or migration requires the formation of a cell front and back. In many cases, polarity occurs in the absence of spatial cues. That is, the cell undergoes symmetry breaking. Understanding the molecular mechanisms that allow cells to break symmetry and polarize requires computational models that span multiple spatial and temporal scales. Here, we apply a multiscale modeling approach to examine the polarity circuit of yeast. In addition to symmetry breaking, experiments revealed two key features of the yeast polarity circuit: bistability and rapid dismantling of the polarity site following a loss of signal. We used modeling based on ordinary differential equations (ODEs) to investigate mechanisms that generate these behaviors. Our analysis revealed that a model involving positive and negative feedback acting on different time scales captured both features. We then extend our ODE model into a coarse-grained reaction–diffusion equation (RDE) model to capture the spatial profiles of polarity factors. After establishing that the coarse-grained RDE model qualitatively captures key features of the polarity circuit, we expand it to more accurately capture the biochemical reactions involved in the system. We convert the expanded model to a particle-based model that resolves individual molecules and captures fluctuations that arise from the stochastic nature of biochemical reactions. Our models assume that negative regulation results from negative feedback. However, experimental observations do not rule out the possibility that negative regulation occurs through an incoherent feedforward loop. Therefore, we conclude by using our RDE model to suggest how negative feedback might be distinguished from incoherent feedforward regulation.

## 1. Introduction

**Figure 1:**
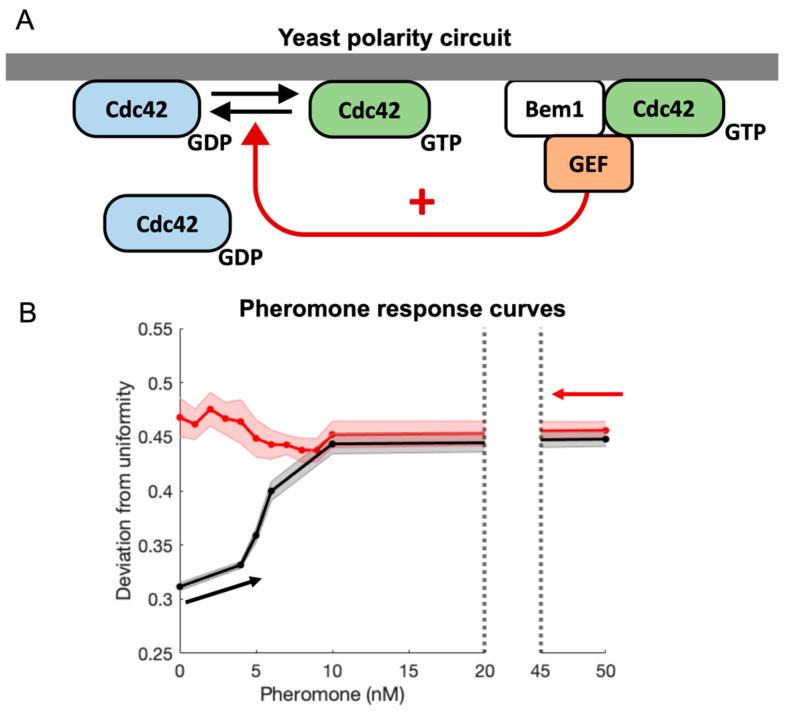
Schematic diagram of the Cdc42 positive feedback loop and pheromone response curves. (**A**) Polarity is driven by a positive feedback loop that generates active Cdc42-GTP at the plasma membrane. Membrane-associated active Cdc42 recruits the GEF Cdc24 in a complex with the polarity scaffold Bem1. This complex promotes the activation of additional Cdc42. (**B**) Pheromone response curves summarizing bistability in the yeast polarity circuit. The curves are derived from the experimental data in Vered 2018 as described in the Methods section. The DfU as measure of polarity is discussed in the Readme file posted at https://datadryad.org/stash/dataset/doi:10.5061/dryad.4f4qrfjn0. The plots demonstrate that at low pheromone concentrations, both the unpolarized (DfU 0.32–0.35) and polarized (DfU ≥ 0.4) steady states are stable, and which state is observed depends on the prior history of the cell’s exposure to pheromones: increasing (black) and decreasing (red) concentrations.

Cells are constantly responding to changes in their environment [1,2,3]. Often, the proper response to an environmental cue requires the generation or reorganization of internal structures. For example, yeast cells undergo directed growth in response to gradients of mating pheromone [4,5,6]. This chemotropic growth requires the formation of an internal structure referred to as the polarity site [7]. The first step in the formation of the polarity site is the establishment of a localized region of high Cdc42 concentration at the cell periphery [8,9,10,11]. Cdc42 is a member of the Rho family of GTPases and functions as a molecular switch, existing in either an inactive or active state [12,13,14]. In its active state, Cdc42 is located in the cell membrane and recruits actin cables along which vesicles are trafficked. The insertion of these vesicles into the cell membrane generates growth in the direction of the polarity site. The biochemical reaction network that generates clusters of Cdc42 activity is referred to as the polarity circuit (Figure 1A). Polarity establishment occurs via a positive feedback loop in which active Cdc42 recruits its activator, the GEF Cdc24, in complex with the scaffold Bem1 from the cytosol. In turn, Cdc24 activates neighboring Cdc42 molecules. This positive feedback loop, coupled with the different diffusion rates associated with membrane-bound active Cdc42 (slow diffusion) and cytosolic inactive Cdc42 (rapid diffusion), is sufficient to generate a polarity site through a Turing mechanism [15]. The ability of the yeast polarity circuit to undergo Turing instability was first demonstrated through mathematical modeling [16]. Since then, mathematical modeling has been used to explain many properties of the polarity circuit.

The kinetics of biochemical networks can be modeled using rate equations that take the form of ordinary differential equations (ODEs) [17,18,19,20]. ODEs represent a coarse-grained description of the system, are computationally efficient to simulate, and often lend themselves to systematic analysis. However, they do not allow for spatial variations in concentrations, which are critical for understanding cell polarity. Therefore, the most common way to model the polarity circuit is using reaction–diffusion equations (RDEs) that take the form of partial differential equations [21,22,23,24,25]. RDEs capture both the biochemical reactions involved in the polarity circuit and the thermal diffusion of molecules. The drawbacks of using RDEs are that they can be computationally expensive to simulate, and they do not capture fluctuations arising from the stochastic nature of biochemical reactions. The dynamics of biochemical networks are most accurately captured by particle-based models [26,27,28]. In these models, every molecule in the system is considered. Molecules undergo thermal diffusion and can react if they are sufficiently close to a reaction partner. These simulations account for stochastic effects due to diffusion and the inherently random nature of chemical reactions. The drawbacks of particle-based models are that they are computationally expensive to simulate and do not lend themselves to theoretical analysis.

We apply each modeling approach to investigate key properties of the yeast polarity circuit across spatial scales of increasing resolution. Yeast cells undergo polarized growth in two different contexts: budding and mating [29,30,31,32]. Polarization during mating requires the same positive feedback circuit that is crucial for polarization during budding. In contrast to budding, polarity establishment during mating requires an external stimulus, mating pheromone, that can easily be manipulated under laboratory conditions. Using an ODE model of the pheromone-induced polarity circuit, we demonstrate that adding negative regulation to the core polarity circuit is sufficient to capture several dynamic properties of yeast polarity establishment, including bistability and the rapid disassembly of the polarity site following the removal of pheromone. We then converted the ODE model to an RDE model by adding terms that account for diffusion. After verifying that the RDE model performs as expected, we extended the model to more faithfully capture the biochemical reactions involved in the polarity circuit. Finally, we recast our detailed RDE model as a particle-based model to demonstrate it still performs as expected when stochastic effects due to the random nature of biochemical reactions are considered. All our models assume that negative regulation of the polarity site occurs through negative feedback. However, it is also possible that an incoherent feedforward loop underlies the rapid dismantling of the polarity site. Therefore, we end by performing an analysis of the RDE model to demonstrate how, in theory, negative feedback can be distinguished from incoherent feedforward regulation.

## 2. Methods

### 2.1. Experimental Datasets

Files containing the experimental data referenced in this manuscript and a document containing an index for the datasets and an explanation of the image analysis methods used to quantify polarity are available on dryad (https://doi.org/10.5061/dryad.4f4qrfjn0).

### 2.2. Dose Response Curves Derived from Experimental Data

The polarity status as pheromone concentration decreases from 50 nM to 0 nM was derived from the dataset indexed as Experiment 7 (https://doi.org/10.5061/dryad.4f4qrfjn0). The average deviation of uniformity (DfU) for the three time points at each pheromone concentration was calculated. The averages and standard error were calculated from five independent experiments. The polarity status as pheromone concentration increases from 0 nM to 50 nM was derived from datasets indexed as Experiments 1–5 (https://doi.org/10.5061/dryad.4f4qrfjn0) as follows. The average DfU for 0 nM and 0 to 4 nM pheromone concentrations were calculated for time points across the 55 min and 70 min time course, respectively, because polarization was not achieved (DfU < 0.35). The average DfU for 6–50 nM was calculated from all time points in the 120 min time course after a polarized status was achieved (DfU > 0.4). The average DfU for 5 nM pheromone was calculated from the last seven time points in the time course. The averages and standard error were calculated from three independent experiments.

### 2.3. Reaction–Diffusion Equation Simulations

Both the simplified and detailed RDE models were solved in 1D using periodic boundary conditions. To generate the time series presented in the figures, we numerically solved the equations using a forward Euler method with a domain length of 2π·2.5 μm. The equations were solved using a spatial step size of ∆x=0.0628μm. The time step was initially set to Δt=0.01 s and reduced until the solution no longer depended on Δt. The bifurcation diagrams for RDE models were generated by simulating the system starting from (1) homogeneous initial conditions to which a small amount of noise was added and (2) a localized initial spike of Cdc42 activity. Following the simulations, the maximum value of active Cdc42 concentration was recorded for each initial condition and used to determine boundaries between the monostable (low active Cdc42 for both initial conditions), bistable (low active Cdc42 for the uniform initial condition and high for the spike initial condition), and Turing unstable (high active Cdc42 for both initial conditions) regimes. To create the bifurcation diagrams, we solved the equations using the MATLAB (R2023b) solver *pdepe* with no-flux boundary conditions and a domain size of π·2.5μm. At a steady state, a solution with periodic boundary conditions can be constructed by reflecting the no-flux solution around the boundary where polarity occurred. We selected several parameter sets to verify that the two simulations methods produced identical steady state solutions.

Parameters for the simplified model are listed in Table S1, and those for the detailed model are listed in Table S2. Most reaction rates for the detailed RDE model were scaled from those for the 2D RDE model in Pablo et al. [28]. All code is available at https://github.com/guanhighyun/yeast-polarity-negative-feedback (accessed on Aug 13 2024).

### 2.4. Particle-Based Simulations

Particle-based simulations were performed using Smoldyn (v2.67) on a Linux-based computing system (Longleaf cluster at UNC, Chapel Hill, NC, USA) [27]. The code is available at https://github.com/guanhighyun/yeast-polarity-negative-feedback (accessed on 13 August 2024). Molecules were regarded as point particles and conducted Brownian motion according to the Euler–Maruyama method. We used a 2D domain with length = $\sqrt{4\pi 2.5^{2}}\approx 8.8623\;\mu m$ and periodic boundary conditions for all simulations. Rates for first-order and second-order inter-particle reactions are listed in Table S3. All of the parameters referred to a previous publication [28] except for GAP-associated reactions ($\lambda_{6a}$, $\lambda_{6b}$, and $k_{6c}$). Polarity strength was measured using Ripley’s K-function (K) [28,33]. K=0 when particles are distributed uniformly. A high K value (K>1.5) signifies strong polarization.

## 3. Results

### 3.1. Experimental Observations

Data demonstrating that the yeast polarity circuit is bistable with respect to pheromone concentration was presented by Vered [34]. These observations were made using a microfluidics chamber to precisely control the temporal profile of pheromone and live-cell imaging of fluorescent reporters to monitor the polarity process in single living cells. Details of the microfluidic device used in these experiments have been published elsewhere [35,36,37]. Here, we summarize observations from three sets of experiments that provided insights into the circuit that were foundational for our model development. (The associated datasets along with an overview of the experiments, description of the experimental methods, and image analysis techniques used to quantify polarity are published on DRYAD: https://doi.org/10.5061/dryad.4f4qrfjn0).

One set of experiments monitored polarity establishment upon exposure to pheromone concentrations of 4, 5, 6, 10, and 50 nM. Analysis of these datasets showed that 6 nM pheromone is the minimum concentration required to establish and maintain a stable polarity patch (See Figure 3.1A–C of [34]). Another notable observation was that at 5 nM pheromone, some cells failed to polarize, but others established a weak and unstable polarity patch that disassembled and reassembled repeatedly throughout the experiment (See Figure 3.1B of [34] and Movie S1).

In another experiment, polarity was established by exposing cells to 50 nM pheromone for 2 h, dropping the concentration to 10 nM (where polarity is stable), and then decreasing the concentration to 0 in 1 nM increments (See Figure 3.2A of [34]). Under this regimen, most cells continued to hold polarity at concentrations below the minimum pheromone concentration required for naïve cells to establish polarity, with some cells maintaining polarity even after the pheromone was completely removed.

Dose–response curves from these two datasets are shown in Figure 1B (see Methods for details for how these curves were constructed and https://doi.org/10.5061/dryad.4f4qrfjn0 for a discussion of using the deviation from uniformity (DfU) as a measure of polarity). The curves demonstrate that polarization as a function of pheromone concentration depends on the history of the system. There is a range of pheromone concentrations for which two steady states co-exist (i.e., the system is bistable). How the system is prepared determines which of these two steady states is experimentally observed (i.e., the system exhibits hysteresis). If we start from low levels of pheromone and increase the concentration, the system will be in the unpolarized state of the bistable regime (Figure 1B, black curve). Alternatively, if we start from high levels of pheromone and gradually reduce the concentration, the system will be in the polarized state of the bistable regime (Figure 1B, red curve). Another way to state this hysteretic effect is that the minimum pheromone concentration required to establish polarity is greater than the minimum pheromone concentration required to maintain polarity. Such hysteretic behavior is a hallmark of bistability.

In another series of experiments, cells were exposed to a high pheromone concentration (50 nM) to establish polarity, and then the pheromone level was abruptly reduced to 0, 4, 5, 6, or 10 nM. Polarity was lost within 5 min when the pheromone was reduced to 5 nM or less (See Figure 3.3A,B of [34]). The same polarity loss occurs for cells exposed to intermediate pheromone concentrations (10 nM) before the pheromone level is abruptly dropped to 0 (see Figure 3.5B of [34]). By contrast, cells polarized by exposure to intermediate pheromone levels (10 nM) can maintain the polarity patch if the pheromone is not completely removed but abruptly dropped to 5 nM (see Figure 3.5B of [34]).

### 3.2. Mechanistic Considerations for Model Development

**Figure 2:**
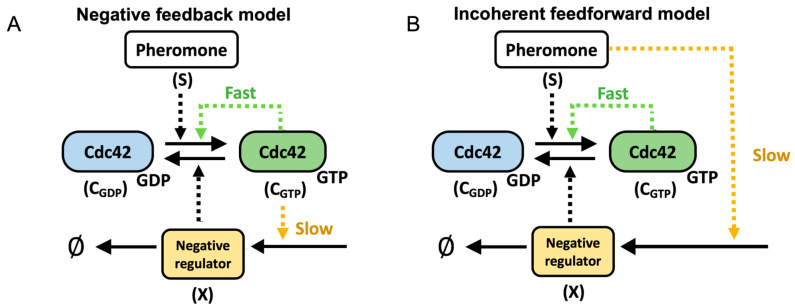
Models for negative feedback and incoherent feedforward regulation. (**A**) For the negative feedback model, the level of the negative regulator X is controlled by the amount of active Cdc42. (**B**) In the case of incoherent feedforward regulation, the level of X is controlled by the pheromone concentration and not affected by active Cdc42.

We reasoned two mechanisms could account for the rapid loss of polarity. In the first scenario, inhibition in the form of either negative feedback (Figure 2A) or incoherent feedforward regulation (Figure 2B) dismantles the polarity site following the removal of the pheromone. In the second mechanism, the slow reinforcement of the polarity site acts to stabilize the site over time. In this scenario, polarity is lost when insufficient time has passed for reinforcement to occur before the pheromone is removed. The reinforcement mechanism is motivated by the observation that actin cables are required for long-term maintenance of the polarity site but not its initial formation [38]. However, our experimental observation that a drop from 10 to 5 nM pheromone levels does not cause a loss of polarity argues against this scenario. Reinforcement would be the same or diminished compared with the drop from 50 to 5 nM, where polarity is destroyed. Therefore, we focus on the negative regulation mechanism. To test if negative regulation is sufficient to explain our experimental results, we developed a series of models of increasing spatial resolution. We focus on negative feedback as the regulatory mechanism responsible for dismantling the polarity site but note that currently available data do not allow us to rule out incoherent feedforward regulation. We end our analysis by suggesting an experiment that might distinguish the two negative regulation mechanisms.

### 3.3. Ordinary Differential Equation Model

The observed rapid disassembly of the polarity patch following the removal of the pheromone suggests the presence of pheromone-induced negative regulation that remains active after the pheromone is removed. In particular, it implies that following pheromone removal, this negative regulation is strong enough to disrupt the positive feedback loop that establishes polarity. By contrast, when the pheromone level is reduced slowly, our results suggest that positive and negative regulation remain balanced so that polarity is maintained. As an initial test of these ideas, we developed an ODE model that consists of a positive feedback loop that generates bistability and a slow negative feedback loop. In the model, Cdc42 exists in an active GTP bound state ($C_{GTP}$) or an inactive GDP bound state ($C_{GDP}$). We assume high levels of $C_{GTP}$ represent a polarized state. We also assume that the total amount of Cdc42 (T) is constant, so that $C_{GDP}=T-C_{GTP}$. The equation that governs the time evolution of $C_{GTP}$ takes the following form:

(1) $$\frac{dC_{GTP}}{dt}=f\left(C_{GTP},X\right)=\left(k_{0}+k_{1}C_{GTP}^{2}\right)\left(T-C_{GTP}\right)-\left(k_{2}+k_{3}X\right)C_{GTP}$$

The first term on the right-hand side in the above equation models the conversion of inactive Cdc42 to its active form. The parameter $k_{0}$ is the basal activation rate. The term $k_{1}C_{GTP}^{2}$ models the positive feedback loop in which active Cdc42 promotes the activation of inactive Cdc42. Biologically, this term represents the recruitment of the GEF Cdc24 by active Cdc42. We assume that the effect of pheromone is to increase this rate: $k_{1}=k_{1}^{0}+s$, where $k_{1}^{0}$ is the basal (pheromone independent) rate and s represents the increase in this rate in the presence of the pheromone. This would occur, for example, if pheromone-induced MAPK activity increased the rate at which the GEF for Cdc42 is recruited to the membrane. The second term in the above equation represents the rate at which active Cdc42 becomes inactive. The parameter $k_{2}$ is the basal rate of inactivation, and the term $k_{3}X$ models the effect of an (as yet) unidentified negative regulator (X). We assume the equation that governs the time evolution of the negative regulator is given by the following:

(2) $$\frac{dX}{dt}={g\left(C_{GTP},X\right)=k}_{4}C_{GTP}-k_{5}X$$

The first term on the right-hand side of the above equation represents the rate at which X increases in response to active Cdc42 ($C_{GTP}$), and the second term represents the rate at which the negative regulator (X) is lost. Our model represents a well-stirred version of the model considered by Chiou et al. [39] to study the competition and coexistence of polarity sites with the additional feature of a negative feedback loop.

**Figure 3:**
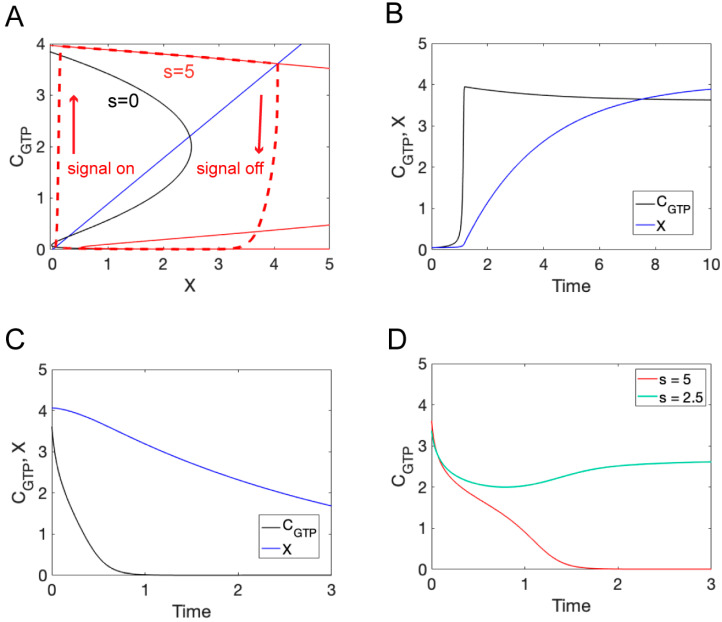
ODE model for negative feedback model. (**A**) Phase-plane for the model: X nullcline (blue), $C_{GTP}$ nullcline for low pheromone concentration (black), and $C_{GTP}$ nullcline for high pheromone concentration (red). Trajectories for $C_{GTP}$ versus X for high pheromone concentration (dashed red curve labeled “signal on”) and when the pheromone concentration is dropped to a low level (dashed red curve labeled “signal off”). (**B**) Time series for $C_{GTP}$ (black) and X (blue) following exposure to a high pheromone concentration. (**C**) Time series for $C_{GTP}$ (black) and X (blue) following the removal of pheromone. (**D**) Comparison of $C_{GTP}$ time series following a drop in pheromone concentration starting from different initial concentrations (s=5, red curve and s=2.5, green curve).

For bistability, Equations (1) and (2) must admit multiple steady-state solutions. A steady state refers to the condition in which $C_{GTP}$ and X are constant in time. That is, $dC_{GTP}/dt=dX/dt=0$. Therefore, to find the steady state values of $C_{GTP}$ and X, we solve the following set of equations: $f(C_{GTP},\;X)=0$ and $g(C_{GTP},\;X)=0$. The two equations define curves in the $X-C_{GTP}$ phase plane referred to as nullclines (Figure 3A). The steady states of the system occur where the nullclines intersect. When s=0 (no pheromone), there are three steady states shown in Figure 3A as intersection of the blue line (X-nullcline) and black curve ($C_{GTP}$-nullcline). The intersection points at low and high values of $C_{GTP}$ represent the unpolarized and polarized states of the system, respectively. The steady state at the intermediate value of $C_{GTP}$ is unstable and cannot be observed experimentally. Therefore, in the absence of pheromone, the system is bistable. When s is increased beyond a critical point, the lower two steady states are lost, and the system transitions to a monostable polarized state, shown in Figure 3A as the intersection of the blue line (X nullcline) and the solid red curve ($C_{GTP}$ nullcline labeled with “s=5”).

To investigate the system’s temporal response to pheromone, we start with s=0 and $C_{GTP}$ and *X* at their steady-state values in the unpolarized state. We then turn on pheromone by setting $s=s_{H}=5$ and numerically solve Equations (1) and (2) (Figure 3B). The concentration of $C_{GTP}$ (active Cdc42) rapidly increases as the system polarizes. This rapid rise in $C_{GTP}$ is followed by a gradual decline as the level of X (negative regulator) increases toward its steady-state value. To gain further insight into the behavior of the system, we can plot $C_{GTP}\left(t\right)$ versus X(t) in the $X-C_{GTP}$ plane (Figure 3A, dashed red curve labeled “signal on”). As can be seen from this graph, $C_{GTP}$ rapidly approaches the $C_{GTP}$ nullcline for $s=s_{H}$ and then follows this curve to the fixed point as X slowly increases. We next use Equations (1) and (2) to simulate the removal of pheromone by starting the model from the steady state for $s=s_{H}=5$ and then setting s=0 (Figure 3C). In this scenario, $C_{GTP}$ rapidly decreases, indicating a loss of polarity, while the value of X decreases over a longer time scale. Again, plotting $C_{GTP}\left(t\right)$ versus X(t) in the $X-C_{GTP}$ plane, we see that $C_{GTP}$ rapidly approaches the lower branch of the s=0 nullcline, before X significantly changes (Figure 3A, dashed red curve labeled “signal off”). The system then follows this nullcline to the unpolarized steady state as X (negative regulator) slowly decreases. Finally, we simulated a drop in pheromone to a level of $s=s_{L}=1$ from two different starting concentrations (Figure 3D). When the initial value of s was taken to be $s_{H}=5$ the system lost polarity when s was dropped to $s_{L}$ (Figure 3D, red curve). However, when the initial value of $s_{I}=2.5$ was used, polarity was maintained following the drop to $s_{L}$ (Figure 3D, green curve).

These results provide evidence in support of a bistable system coupled with slow negative feedback. However, the ODE model does not consider spatial aspects of polarity establishment, and therefore, it is not clear if the results still apply when spatiotemporal models are considered.

### 3.4. Reaction–Diffusion Equation Models

Next, we updated our model to include spatial variations in the concentrations of the molecular species in the polarity circuit. To start our analysis, we considered a model without negative feedback:

(3) $$\frac{\partial C_{GTP}}{\partial t}=\left[\left(k_{0}+k_{1}C_{GTP}^{2}\right)C_{GDP}-k_{2}C_{GTP}\right]+D_{m}\frac{\partial^{2}C_{GTP}}{{\partial x}^{2}}$$

(4) $$\frac{\partial C_{GDP}}{\partial t}=-\left[\left(k_{0}+k_{1}C_{GTP}^{2}\right)C_{GDP}-k_{2}C_{GTP}\right]+D_{c}\frac{\partial^{2}C_{GDP}}{{\partial x}^{2}}$$

where $C_{GTP}$ is localized to the membrane and $C_{GDP}$ is located in the cytosol. The last terms in Equations (3) and (4) account for the effects of diffusion. Importantly, diffusion in the membrane is substantially slower than diffusion in the cytosol (i.e., $D_{m}\ll D_{c}$).

**Figure 4:**
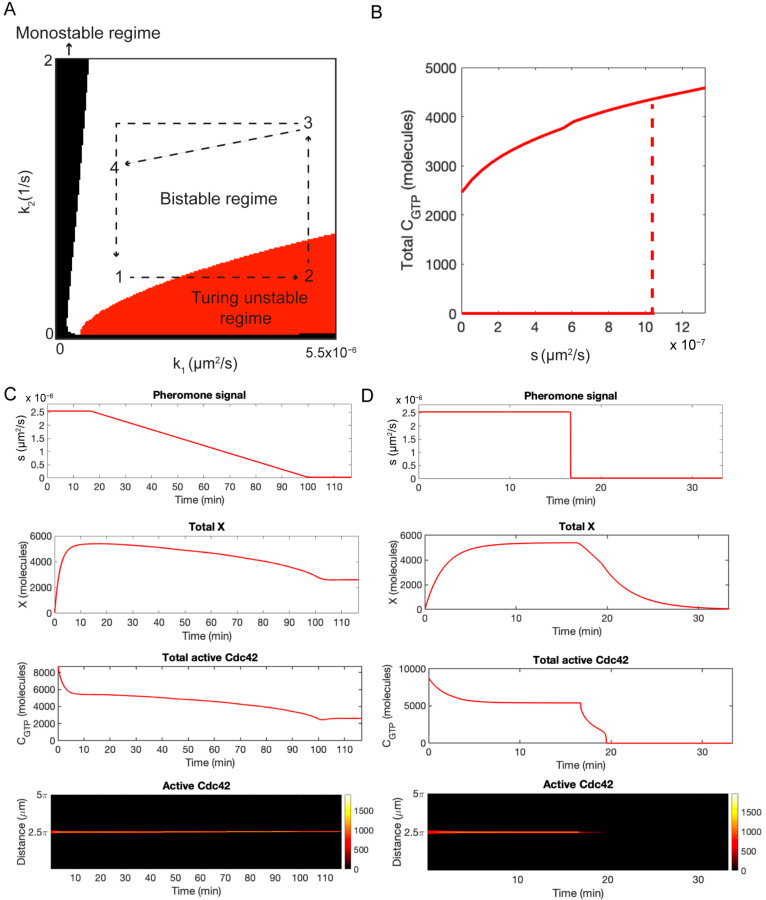
Results for the simplified negative feedback model. (**A**) A two-parameter bifurcation diagram for the core polarity model without negative feedback. The rate constant $k_{1}$ governs the strength of the positive feedback, and $k_{2}$ is the rate constant for the deactivation of Cdc42. The black arrows illustrate the NFB model path through the phase space for the two different experiments. (**B**) A single parameter bifurcation diagram for the negative feedback model in terms of the signal s. The red lines indicate the total amount of active Cdc42. The transition from the bistable to Turing unstable regime occurs at $s=1.05\times 10^{-6}$ μm^2^/s. (**C**) Time series for the case in which stimulus is slowly ramped down. At t=0, the signal is turned on ($s=2.54\times 10^{-6}$ μm^2^/s). It remains constant until t=16.67 min, at which time s is slowly ramped down to 0 (top panel). The total amount of the negative regulator X increases slowly following exposure to the stimulus and remains elevated during the ramp down of s (middle panel). Polarity is established rapidly following exposure to the stimulus and is retained as s is ramped down (bottom panel). (**D**) Same as (**C**) except s is set to 0 at t=16.67 min (top panel). In this case, the polarity site is rapidly lost following the removal of the stimulus (bottom panel), and the total amount of X decreases (middle panel). Model parameters are as specified in Table S1.

To begin our analysis, we constructed a two-parameter bifurcation diagram using the rate constants $k_{1}$ (positive feedback) and $k_{2}$ (deactivation) to characterize the steady-state behavior of Equations (3) and (4) (Figure 4A). This diagram consists of three regions. In the bistable region (white area), the spatially homogenous and polarized steady states are stable to small perturbations. In the Turing unstable region (red area), the spatially homogenous solution is unstable, and the system always evolves to a polarized state. In the monostable region (black area), the system does not polarize, and the only steady-state solution is spatially homogenous. As we demonstrate below, when negative regulation is added to the model, we can interpret the behavior of the extended model in terms of movement through the parameter space.

Next, we update the model equations to include the effect of pheromone and a negative feedback loop (Figure 2A). The revised model equations are as follows:

(5) $$\frac{\partial C_{GTP}}{\partial t}=\left(k_{0}+\left(k_{1}^{0}+s\right)C_{GTP}^{2}\right)C_{GDP}-\left(k_{2}^{0}+k_{3}X\right)C_{GTP}+D_{m}\frac{\partial^{2}C_{GTP}}{{\partial x}^{2}}$$

(6) $$\frac{\partial C_{GDP}}{\partial t}=-\left(k_{0}+\left(k_{1}^{0}+s\right)C_{GTP}^{2}\right)C_{GDP}+\left(k_{2}^{0}+k_{3}X\right)C_{GTP}+D_{c}\frac{\partial^{2}C_{GDP}}{{\partial x}^{2}}$$

(7) $$\frac{\partial X}{\partial t}{=k}_{4}C_{GTP}-k_{5}X+D_{c}\frac{\partial^{2}X}{{\partial x}^{2}}$$

Again, we assume that the effect of the pheromone is to increase the strength of the positive feedback loop through the relation $k_{1}=k_{1}^{0}+s$. We replace $k_{2}$ with the expression $k_{2}=k_{2}^{0}+k_{3}X$, where X is the concentration of the negative regulator. In Equation (7), we assume that the production rate of X depends on $C_{GTP}$, forming a negative feedback loop. We also assume that X exists in the cytosol and, therefore, diffuses at a rate governed by $D_{c}$.

We are now able to use the bifurcation diagram to understand the behavior of the model with negative feedback. In the absence of pheromone, the system starts in the bistable regime in the unpolarized steady state (Figure 4A, position 1). The addition of pheromone increases $k_{1}$ and, therefore, moves the system into the Turing unstable regime, leading to polarization (Figure 4A, position 2). Next, there is a slow buildup of X that increases $k_{2}$ (Figure 4A, position 3). If s is reduced slowly, the activation rate, $k_{0}+\left(k_{1}^{0}+s\right)C_{GTP}^{2}$, and deactivation rate, $k_{2}^{0}+k_{3}X$, remain balanced, and the system stays polarized after pheromone is completely removed (Figure 4A, position 4). However, if s is reduced rapidly to zero, the activation rate drops immediately, but the delayed response of the negative feedback loop allows X to remain elevated long enough to destroy polarity, and the system returns to the spatially homogenous steady state (Figure 4A, position 1).

To support this qualitative explanation, we performed computer simulations of the RDE model given by Equations (5)–(7) in a 1D domain using periodic boundary conditions. Full details for the numerical methods are provided in the Methods section. We first constructed a one-parameter bifurcation diagram using s as the bifurcation parameter (Figure 4B). Consistent with our experimental results (Figure 1B), the model is bistable when s=0 and undergoes a Turing bifurcation at $s=1.05\times 10^{-6}\;{\mu m}^{2}/s$. Next, we simulated the case in which the system is started in the Turing unstable regime ($s=2.54\times 10^{-6\;}{\mu m}^{2}/s$), and s is slowly decreased in time starting at t=16.7 min. (Figure 4C). As can be seen, both the active Cdc42 concentration, $C_{GTP}$, and X concentration have time to respond to the slowly changing signal, and polarity is maintained. In the second simulation, we used the same initial condition ($s=2.54\times 10^{-6}\;{\mu m}^{2}/s$). However, this time, the signal was turned off at t=16.7 min (Figure 4D). In this scenario, the polarity site is dismantled after the signal is removed.

### 3.5. Detailed RDE Model

**Figure 5:**
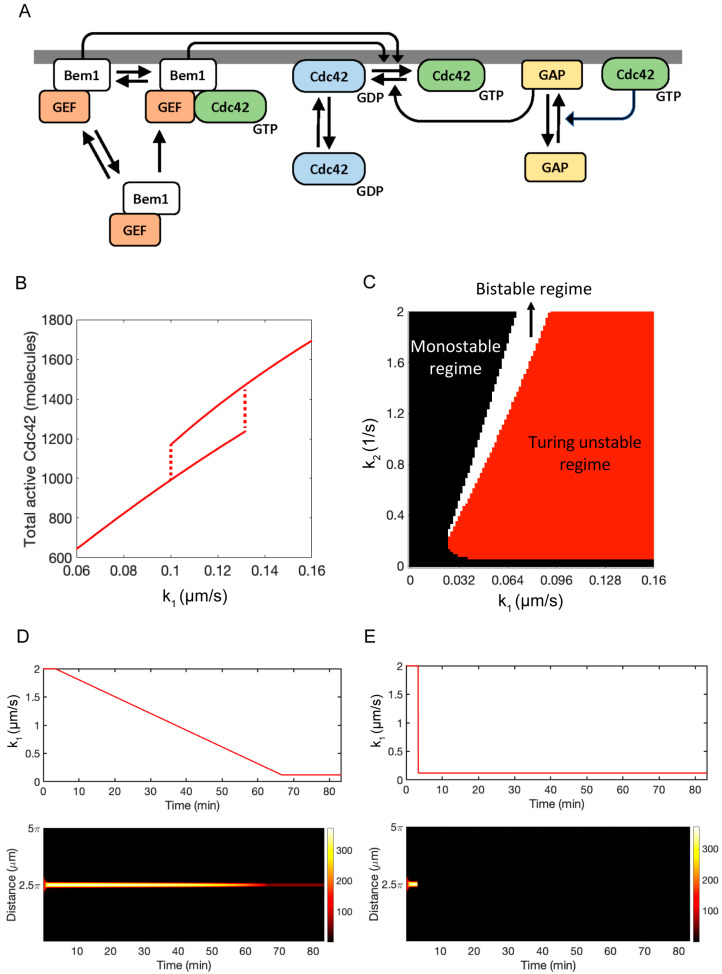
A detailed RDE model for negative feedback regulation. (**A**) Reaction scheme for the detailed RDE model. (**B**) A single parameter bifurcation diagram in terms of the positive feedback strength $k_{1}$. (**C**) A two-parameter bifurcation diagram in terms of positive feedback ($k_{1}$) and Cdc42 deactivation ($k_{2}$). (**D**) Time series for active Cdc42 distribution (bottom panel) after gradually decreasing positive feedback (top panel). (**E**) Same as (**D**) except that $k_{1}$ is reduced at one step.

While the model above captures qualitative properties of the polarity circuit, it lacks many of the biochemical steps involved in the system. To test if a more accurate representation of the system would behave in a similar fashion, we started with the model and parameter values presented by Pablo et al. [28]. To this model, we added a negative feedback loop in which active Cdc42 increases the rate at which its GAP gets activated. A schematic diagram of the extended model is shown in Figure 5A. This system can be represented with the following set of reaction–diffusion equations:

(8) $$\frac{\partial {Cdc42D}_{c}}{\partial t}=D_{c}\nabla^{2}{Cdc42D}_{c}+k_{5b}{Cdc42D}_{m}-k_{5a}{Cdc42D}_{c}$$

(9) $$\frac{\partial {Cdc42D}_{m}}{\partial t}=D_{m}\nabla^{2}{Cdc42D}_{m}+\left(k_{2}+k_{3}{GAP}_{a}\right)Cdc42T+k_{5a}{Cdc42D}_{c}\;-\left(k_{10}{GEF}_{m}+k_{1}\left[{GEF}_{m}Cdc42T\right]+k_{5b}\right){Cdc42D}_{m}$$

(10) $$\frac{\partial Cdc42T}{\partial t}=D_{m}\nabla^{2}Cdc42T+\left(k_{10}{GEF}_{m}+k_{1}\left[{GEF}_{m}Cdc42T\right]\right){Cdc42D}_{m}+k_{9b}\left[{GEF}_{m}Cdc42T\right]\;-\left(k_{2}+k_{9a}{GEF}_{m}+k_{7}{GEF}_{c}+k_{3}{GAP}_{a}\right)Cdc42T$$

(11) $$\frac{\partial {GEF}_{c}}{\partial t}=D_{c}\nabla^{2}{GEF}_{c}+k_{8b}{GEF}_{m}-\left(k_{8a}+k_{7}Cdc42T\right){GEF}_{c}$$

(12) $$\frac{\partial {GEF}_{m}}{\partial t}=D_{m}\nabla^{2}{GEF}_{m}+k_{8a}{GEF}_{c}+k_{9b}\left[{GEF}_{m}Cdc42T\right]-\left(k_{8b}+k_{9a}Cdc42T\right){GEF}_{m}$$

(13) $$\frac{\partial {[GEF}_{m}Cdc42T]}{\partial t}=D_{m}\nabla^{2}[{GEF}_{m}Cdc42T]+\left(k_{9a}{GEF}_{m}+k_{7}{GEF}_{c}\right)Cdc42T-k_{9b}\left[{GEF}_{m}Cdc42T\right]$$

(14) $$\frac{\partial {GAP}_{i}}{\partial t}=D_{c}\nabla^{2}{GAP}_{i}+k_{6}{GAP}_{a}-k_{4}Cdc42T{\;GAP}_{i}$$

(15) $$\frac{\partial {GAP}_{i}}{\partial t}=D_{c}\nabla^{2}{GAP}_{i}+k_{6}{GAP}_{a}-k_{4}Cdc42T{\;GAP}_{i}$$

All the parameter values are given in Table S2.

We first verified that the system produces bistable behavior as the pheromone concentration (implicitly modeled in the positive feedback rate $k_{1}$) is increased (Figure 5B). We then generated a two-parameter bifurcation diagram for the system lacking negative feedback ($k_{3}=0$) as performed above for the simpler model (Figure 5C). The qualitative features of the bifurcation diagram remained unchanged for the extended model. We verified that the full system would maintain polarity if the pheromone concentration was reduced slowly and lose polarity if pheromone was rapidly removed (Figure 5D,E). Overall, results from our detailed RDE model align closely with the outcomes of the simplified model, effectively encapsulating yeast polarity features with a more realistic representation of reaction networks.

### 3.6. Particle-Based Model

**Figure 6:**
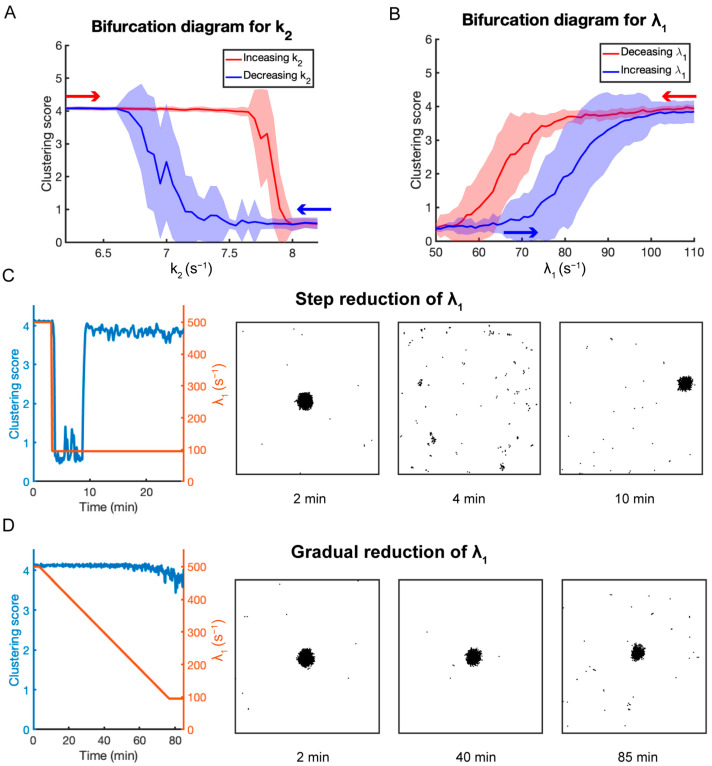
Results for the particle-based model. (**A**) Bifurcation diagram of the clustering score in terms of the Cdc42 deactivation rate ($k_{2}$). The red curve represents results for increasing $k_{2}$ and the blue for decreasing $k_{2}$. Simulations were performed with 5000 Cdc42 and 500 GEF molecules with no negative feedback regulation. (**B**) Bifurcation diagram in terms of the positive feedback rate $\lambda_{1}$ including negative feedback using 1000 GAP molecules. The red curve represents results for decreasing $\lambda_{1}$ and the blue curve for increasing $\lambda_{1}.$ (**C**) Simulation results for a sudden reduction in $\lambda_{1}$. Time series for $\lambda_{1}$ (red curve) and the clustering score (blue curve) (left panel). Distributions of Cdc42-GTP molecules at indicated times (right panels). (**D**) Same as (**C**) except that $\lambda_{1}$ is reduced gradually. Model parameters are specified in Table S3.

While RDE models capture many qualitative features of the yeast polarity circuit, one of their shortcomings is that they do not capture the time scale over which polarity occurs, taking longer to polarize than observed experimentally [28]. Therefore, we turned to particle-based simulations to investigate how molecular-level fluctuations affect the performance of the polarity circuit [28]. We found that these intrinsic fluctuations substantially decrease the time needed for the system to polarize. However, the previous model did not include negative feedback or operate in a bistable regime. Therefore, we converted the detailed RDE model given by Equations (8)–(15) into a particle-based model that we simulated using the software application Smoldyn (v2.67) [27]. We used a cluster score based on Ripley’s K-function as described in the Methods section as a measure of polarity. Similar to the detailed RDE model, the particle-based model of the polarity circuit also showed a bistable region as the Cdc42 deactivation rate $k_{2}$ was varied (Figure 6A). When GAP-mediated negative feedback was added to the polarity circuit, the system continued to display bistable behavior and hysteresis as the positive feedback strength was varied (Figure 6B). To vary the positive feedback strength in the particle-based model, we varied the parameter $\lambda_{1}$, which is the rate at which inactive Cdc42 is converted to active Cdc42 if a Cdc42-GTP-GEF heterodimer is within the reaction radius. Consistent with our experimental observations, suddenly reducing $\lambda_{1}$ destroyed polarity (Figure 6C, Movie S3), while slowly reducing $\lambda_{1}$ allowed polarity to be maintained (Figure 6D, Movie S4). Interestingly, for the case in which the pheromone level is suddenly dropped, and polarity is lost, the polarity site eventually reformed at another location (Figure 6C). We also observed this behavior in a subset of our experiments (Movie S2). The time scale for polarization by the particle-based model was consistent with our experimental observations (~10–70 min). Our particle-based simulations confirm that the results obtained from our RDE simulations remain valid in the presence of molecular-level noise and capture additional features of the experimental results.

### 3.7. Negative Feedback Versus Incoherent Feedforward Regulation

In the models presented above, we assumed that negative feedback (NFB) was responsible for dismantling the polarity site following removal of pheromone. However, negative regulation through an incoherent feedforward (IFF) loop might also underlie this behavior. An incoherent feedforward loop is formed when a pheromone leads to the activation of a negative regulator of Cdc42 independently of Cdc42 activity (Figure 2B). To convert the model to one regulated by an incoherent feedforward loop, the only change we made was to the equation for the negative regulator:

(16) $$\frac{\partial X}{\partial t}{=k}_{4}s-k_{5}X+D_{c}\frac{\partial^{2}X}{{\partial x}^{2}}$$

The rest of the equations remained unchanged, and all parameter values remained the same, except for those in the equation above (see Table S1). In Equation (16), the synthesis rate of the negative regulator X depends directly on the signal strength s, as opposed to the amount of active Cdc42 as in the case of NFB (see Equation (7)). Our previous results from NFB simulations hold true for IFF simulations: (1) the system undergoes a bifurcation from a bistable region to a single steady state as the signal s is increased (Figure S1A); (2) polarity is maintained if the pheromone signal is gradually reduced (Figure S1B) but is lost if the signal is rapidly removed (Figure S1C). The similar behavior of the NFB and IFF models made us wonder if it was, in theory, possible to distinguish these two forms of negative regulation.

For the NFB model, the concentration of the negative regulator X is dependent on the amount of active Cdc42, whereas, for the IFF model, X is independent of Cdc42 and only depends on the pheromone concentration. For both models, in the absence of pheromone (s=0), the system is in a bistable regime and starts from the unpolarized state. When the pheromone level is increased, both systems move into a regime where only the polarized state is stable. If the system is started in this regime and the pheromone level is slowly reduced so that polarity is maintained, the amount of X will be different in the two models. For NFB regulation, X will remain elevated because its level depends on active Cdc42. In contrast, for IFF regulation, X will decrease because, in this model, the level of X depends only on the signal strength and not the amount of active Cdc42. This difference leads to a potential experimental method for distinguishing the two models.

**Figure 7:**
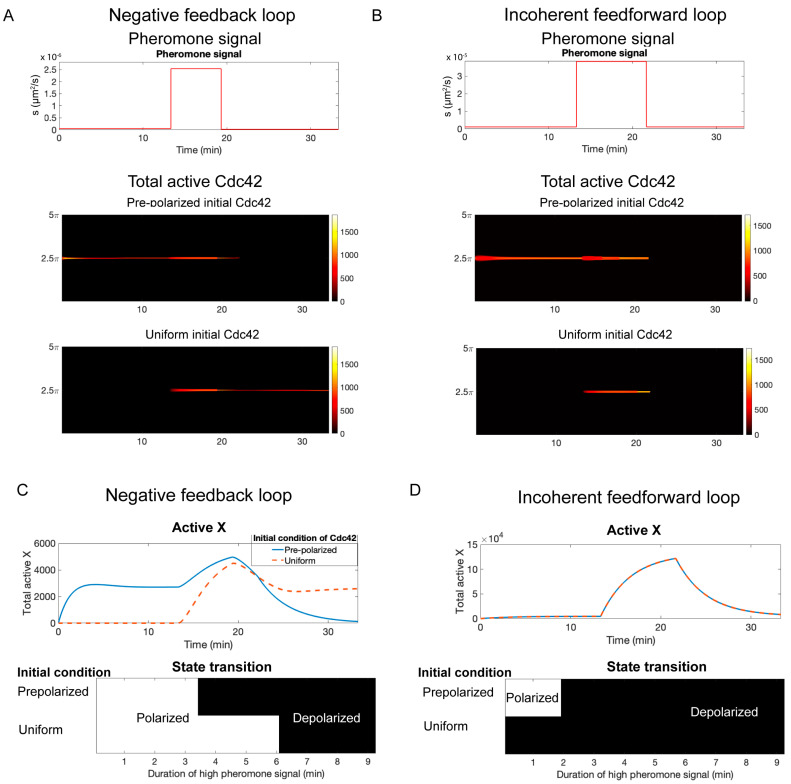
Negative feedback versus incoherent feedforward regulation. (**A**) Results for the NFB model. Time-dependent signal (top panel) and Cdc42-GTP distributions using different initial conditions: polarized (middle panel) and uniform (bottom panel). (**B**) Same as (**A**) for the IFF model. (**C**) The change in the total amount of the negative regulator X (upper panel) and dependence of the final solution on the signal duration (lower panel) for the NFB model. (**D**) Same as (**C**) for the IFF model.

The experiment is to start with no pheromone (s=0) but using two different initial conditions. In the first case, the system starts from an unpolarized state. In the second case, the system is started from the polarized state. Experimentally, this would require exposing cells to high pheromone concentrations, then slowly reducing the concentration to zero. Both initial conditions are exposed to a level of pheromone sufficient to induce polarity. Next, the pheromone is rapidly removed (Figure 7A,B, top panels). For the NFB model, the system that started in the polarized state has a higher amount of X at the time the pheromone concentration is removed (Figure 7C, top panel), and polarity is lost (Figure 7A, middle panel). Depending on the length of time before pheromone is removed, the system that is started in the unpolarized state will maintain polarity (Figure 7A, bottom panel) or, possibly, lose polarity at a slower rate. However, for the IFF model, the amount of X is the same in both scenarios (Figure 7D, top panel) and polarity is lost at the same rate (Figure 7B, middle and bottom panels). If we vary the duration of pheromone exposure, for the NFB model the uniform initial condition requires a longer signal duration than the polarized initial condition for polarity to be lost following removal of pheromone (Figure 7C, bottom panel), whereas the IFF polarity is always lost for the uniform initial condition (Figure 7D, bottom panel).

## 4. Discussion

Cdc42 is a member of the Rho family of GTPases. A primary role for these signaling molecules is the regulation of the actin cytoskeleton. Mathematical models have been used to investigate many aspects of Rho GTPase signaling. The signaling circuit shown in Figure 2A not only underlies polarity establishment [16,40,41,42,43,44,45] but also produces waves and other patterns of signaling activity observed in cellular functions including cell division, cell migration, and phagocytosis [46,47,48,49,50]. The different behaviors arise by varying parameters in the core circuit that governs the strength and timing of feedback regulation. While these systems are typically modeled as mass-conserved reaction diffusion equations, intrinsic fluctuations arising from the stochastic nature of biochemical reactions can significantly impact their behavior. Therefore, understanding the mechanisms that regulate Rho GTPase signaling requires models that can capture multiple spatial and temporal scales.

### 4.1. Multiscale Models of the Polarity Circuit

Our modeling strategy was to start with a coarse-grained ODE model for the core polarity circuit that includes a known positive feedback loop in which Cdc42 recruits its own activator. To this core circuit, we added a negative feedback loop in which Cdc42 activity leads to the activation of a negative regulator. A key feature of the expanded model is that the feedback loops act on different time scales: rapid positive feedback and slow negative feedback. The computational and analytical tractability of the ODE model allowed us to demonstrate that it was sufficient to generate both bistable behavior and the rapid dismantling of the polarity site following the removal of the pheromone. While the ODE model provided evidence to support the role of negative feedback in dismantling the polarity site, it did not consider the spatial aspects of polarity establishment. Therefore, we turned to reaction–diffusion equations. Initially, to construct an RDE model, we simply added diffusion to our ODE model. In the absence of negative feedback this model reduces to the model studied by Chiou et al. [39] to investigate competition and coexistence between polarity sites. The model equations for the RDE model were simple enough that a qualitative understanding of the system’s behavior could be achieved through a combination of simulations and bifurcation analysis. Our analysis demonstrated that the qualitative properties of the system were maintained when spatial variations in concentrations were considered.

We then extended the model to represent the polarity circuit more faithfully by including more of the known chemical reactions in this system. Our extended model is essentially the one considered by Howell et al. [51] to study oscillations in the polarity site. While the extended model again qualitatively captured our experimental observations, the stochastic noise inherent in biochemical reaction and diffusion was not captured by the model. Previously, we used particle-based simulations of the polarity circuit to study the effects of intrinsic fluctuations on the performance of the system. Therefore, we converted the extend RDE model to a particle-based model using the method outlined in [52]. Again, the qualitative features of the system were preserved, and polarization occurred on time scales consistent with our experimental observations.

### 4.2. Negative Feedback Versus Incoherent Feedforward Regulation

Our results do not distinguish if pathway inhibition occurs through negative feedback or incoherent feedforward regulation. The biochemical network responsible for polarity establishment during mating also underlies polarity establishment during budding. There are known negative feedback loops that regulate polarization during budding and, therefore, are also likely to operate during the mating response. These include the reduction in activity of the GEF Cdc24 through feedback phosphorylation [53] and the recruitment of GAPs to the polarity site [54,55,56]. While there is no known incoherent feedforward loop that acts at the level of the polarity circuit, one does exist at the level of transcription regulation [36]. MAPK activity leads to activation of the transcriptional activator Ste12. At the same time, MAPK kinase activity leads to the activation of Far1, a multifunction protein. One function of Far1 is to increase the rate at which Ste12 is degraded [57], thereby forming an incoherent feedforward loop. Far1 is induced by Ste12 and also recruits the GEF Cdc24 to the polarity site. Therefore, the degradation of Ste12 could indirectly have a negative impact on polarity. While our experimental investigations were not sufficient to distinguish these potential mechanisms, recent computational and theoretical investigations have suggested methods for accomplishing this task in the case of ODE models [58]. Therefore, we investigated if a similar strategy could be employed with our models. Rahi et al. were interested in systems that adapt over time (i.e., show a transient response to a sustained signal). There are three key differences between the polarity circuit and the signaling networks considered by Rahi et al. First, the polarity circuit does not show an adaptive response; second, the polarity circuit is a spatially extended system; and third, the polarity circuit is bistable. We took advantage of the third property, bistability, to design an experiment that could, in theory, distinguish the two mechanisms. In the experiment, the system is prepared with two different starting conditions (polarized and unpolarized) and no pheromone present. In the case of NFB, the amount of negative regulator present in the system depends on the starting condition because the strength of negative regulation is tied to the concentration of active Cdc42. In contrast, for IFF regulation, the amount of the negative regulator is the same in both scenarios. Next, a pheromone concentration sufficient to establish polarity is added to both initial conditions. For NFB regulation, the negative regulator starts at an elevated level for the pre-polarized system, while it starts at a low level for the system in the unpolarized state. Therefore, if pheromone is removed soon after polarity is established, the system started from a pre-polarized state will immediately lose polarity. However, the system starting from the unpolarized state will either maintain polarity or lose it at a slower rate than the pre-polarized system. In contrast, for IFF regulation, the amount of the negative regulator will be the same for both starting conditions. Therefore, no difference will be seen when the pheromone is removed.

### 4.3. Potential Benefits of Negative Regulation of a Bistable Circuit

Bistability may provide important benefits during the mating response. Because yeast cells are not motile, they must undergo polarized growth to reach a distant mating partner. This chemotropic growth entails considerable energetic costs and presumably will be undertaken only when there is a high probability of successful mating. Monostable systems that contain strong positive feedback loops are characterized by sigmoidal or switch-like dose–response curves. Therefore, in the region where the polarity circuit transitions from an unpolarized to a polarized state, the system is highly sensitive to noise. Consequently, small fluctuations in the pheromone concentration can cause cells to gain or lose polarity. In contrast, bistable systems are more robust to perturbations and provide a mechanism for guarding against loss of polarity once polarity is established. Additionally, bistable regulation of polarity has also been suggested to improve sensitivity to shallow noisy gradients [59,60].

Our analysis also revealed the existence of negative regulation acting over time scales that are longer than the positive feedback loop driving polarity. Yeast cells often polarize in directions that are not aligned with the pheromone gradient and then reorient the polarity site as they grow. Because positive feedback tends to reinforce the polarity site, negative feedback is likely required to move the site. Indeed, previous studies have shown that vesicle delivery represents a delayed negative feedback loop by locally diluting Cdc42 concentrations [38,61,62] and is required for polarity patch movement. In another study that combined experimental and modeling investigations, it was demonstrated that negative feedback generates oscillatory behavior of the polarity site and increases the robustness of the polarity circuit to fluctuations in molecular abundances [51]. Our results suggest an additional physiological benefit of delayed negative regulation may be to rapidly dismantle the polarity site if the signal is abruptly lost when a competing suitor successfully mates with a nearby potential partner.

In summary, we applied a multiscale modeling approach to investigate feedback regulation in the yeast polarity circuit. Our strategy was to start with a deliberately simplified ODE model to gain insight into the system-level properties of the polarity circuit. Guided by our analysis of this model, we developed a series of spatiotemporal models of increasing biological detail and spatial resolution. While we focused on the yeast polarity circuit, we believe a similar approach can be applied to other intracellular signaling systems.

## Data Availability

Experimental data are available at Polarization of yeast cells under different pheromone induction regimens [Dataset]. Dryad. https://doi.org/10.5061/dryad.4f4qrfjn0.

## Supplementary Materials

The following are available online at https://www.mdpi.com/article/10.3390/cells13161358/s1, Figure S1: Results for the Incoherent feedforward model, Table S1: Parameters for the simplified polarity circuit RDE model, Table S2: Parameters for the detailed polarity circuit model, Table S3: Parameters for the particle-based model, Video S1: Under 5 nM pheromone, the cell established an unstable polarity patch that disassembled and reassembled dynamically, Video S2: The cell quickly depolarized and gradually repolarized when the pheromone concentration was decreased from 50 nM to 5 nM, Video S3: Spatiotemporal distribution of Cdc42-GTP after sudden reduction in the positive feedback rate, Video S4: Spatiotemporal distribution of Cdc42-GTP with slow reduction in the positive feedback rate.

## Abbreviations

DfU	deviation from uniformity
GAP	GTPase activating protein
GEF	guanine nucleotide exchange factor
ODE	ordinary differential equation
RDE	reaction–diffusion equation
NFB	negative feedback
IFF	incoherent feedforward
