# Supplementary material for: Multiscale Modeling of Bistability in the Yeast Polarity Circuit
Source: Cells. 2024 Aug 15;13(16):1358. doi: 10.3390/cells13161358 (PMC11352540; doi:10.3390/cells13161358)
Supplement: Supplementary file 1 [file cells-13-01358-s001.zip › cells-3112440-supplementary.pdf]

Table S1. Parameters for the simplified polarity circuit RDE model.

**Description**	**Parameter**	**Value**
Basal activation rate of Cdc42	k_0	0.1 s^{-1} for core circuit simulations without negative feedback (Fig. 4A).<br>0 for all other simulations in Fig. 4 and Fig. 7.
Positive feedback of Cdc42	k_1^0	6.6224 × 10^{-7} $\mu m^2\ s^{-1}$
Basal inactivation rate of Cdc42	k_2^0	0.14 s^{-1}
Pheromone signal strength	s	Varied ($\mu m^2\ s^{-1}$)
Inactivation of Cdc42 by GAP	k_3	0 for core circuit simulations without negative feedback (Fig. 4A).<br>0.0083 $\mu m\ s^{-1}$ for all other simulations in Fig. 4 and Fig. 7.
Activation of GAP by Cdc42 (in NFB simulations) or pheromone (in IFF simulations)	k_4	0 for core circuit simulations without negative feedback (Fig. 4A).<br>0.01 s^{-1} for NFB simulations in Fig. 4 and Fig. 7.<br>2.1817 x 10^6 μm^{-3} for IFF simulations in Fig. 7.
Inactivation of GAP	k_5	0 for core circuit simulations without negative feedback (Fig. 4A).<br>0.01 s^{-1} for all other simulations in Fig. 4 and Fig. 7.
Total amount of Cdc42	C_{tot}	9456 molecules
Diffusion coefficient in cytoplasm	D_c	0.25 $\mu m^2\ s^{-1}$
Diffusion coefficient on membrane	D_m	0.0025 $\mu m^2\ s^{-1}$
Domain length	l	5 π μm

Table S2. Parameters for the detailed polarity circuit model.

Description	Parameter	Value
Cdc42T-Bem1-GEF + $Cdc42D_m$ → Cdc42T-Bem1-GEF + Cdc42T	k_1	Varied ($\mu m^2 s^{-1}$)
Cdc42T → $Cdc42D_m$	k_2	1 s^{-1}
Cdc42T + GAP_a → $Cdc42D_m$ + GAP_a	k_3	0.16 $\mu m\ s^{-1}$
Cdc42T + GAP_i → Cdc42T + GAP_a	k_4	2×10^{-4} $\mu m\ s^{-1}$
$Cdc42D_c$ → $Cdc42D_m$	k_{5a}	36 s^{-1}
$Cdc42D_m$ → $Cdc42D_c$	k_{5b}	13 s^{-1}
GAP_a → GAP_i	k_6	0.01 s^{-1}
Cdc42T + $BemGEF_c$ → Cdc42T-Bem1-GEF	k_7	0.3695 $\mu m\ s^{-1}$
Bem1-GEF_c → Bem1-GEF_m	k_{8a}	10 s^{-1}
Bem1-GEF_m → Bem1-GEF_c	k_{8b}	40 s^{-1}
Cdc42T + Bem1-GEF_m → Cdc42T-Bem1-GEF	k_{9a} (λ_{9a})	0.0118 $\mu m\ s^{-1}$
Cdc42T-Bem1-GEF → Cdc42T + $Bem1GEF_m$	k_{9b}	34.5262 s^{-1}
Bem1-GEF_m + $Cdc42D_m$ → Bem1-GEF_m + Cdc42T	k_{10}	0.0076 $\mu m\ s^{-1}$
Diffusion coefficient in cytoplasm	D_c	15 $\mu m^2\ s^{-1}$
Diffusion coefficient on membrane	D_m	0.0025 $\mu m^2\ s^{-1}$
Total Cdc42	Cdc42	5000 molecules
Total Bem1-GEF	Bem1-GEF	500 molecules
Total GAP	GAP	1000 molecules

Domain length	l	5 π μm
Time step	Δt	$\leqslant$0.01 s

Table S3. Parameters for the particle-based model.

Description	Parameter	Value
Cdc42T-Bem1-GEF + $Cdc42D_m$ → Cdc42T-Bem1-GEF + Cdc42T	λ_1	180 s^{-1}
Cdc42T → $Cdc42D_m$	k_2	0.35 s^{-1}
Cdc42T + GAP_a → $Cdc42D_m$ + GAP_a	λ_3	110 s^{-1}
Cdc42T + GAP_i → Cdc42T + GAP_a	λ_4	0.15 s^{-1}
$Cdc42D_c$ → $Cdc42D_m$	k_{5a}	36 s^{-1}
$Cdc42D_m$ → $Cdc42D_c$	k_{5b}	13 s^{-1}
GAP_a → GAP_i	k_6	0.01 s^{-1}
Cdc42T + $BemGEF_c$ → Cdc42T-Bem1-GEF	λ_7	256 s^{-1}
Bem1-GEF_c → Bem1-GEF_m	k_{8a}	10 s^{-1}
Bem1-GEF_m → Bem1-GEF_c	k_{8b}	40 s^{-1}
Cdc42T + Bem1-GEF_m → Cdc42T-Bem1-GEF	λ_{9a}	9.6 s^{-1}
Cdc42T-Bem1-GEF → Cdc42T + $Bem1GEF_m$	k_{9b}	40 s^{-1}
Bem1-GEF_m + $Cdc42D_m$ → Bem1-GEF_m + Cdc42T	λ_{10}	5.3 s^{-1}
Diffusion coefficient in cytoplasm	D_c	15 μm^2 s^{-1}
Diffusion coefficient on membrane	D_m	0.0025 μm^2 s^{-1}
Total Cdc42	Cdc42	5000 molecules

Total Bem1-GEF	Bem1-GEF	500 molecules
Total GAP	GAP	1000 molecules
Surface area	A	$4 \times 2.5^2 \pi$ μm^2
Reactive radius	ρ	0.05 μm
Time step	Δt	0.1 ms

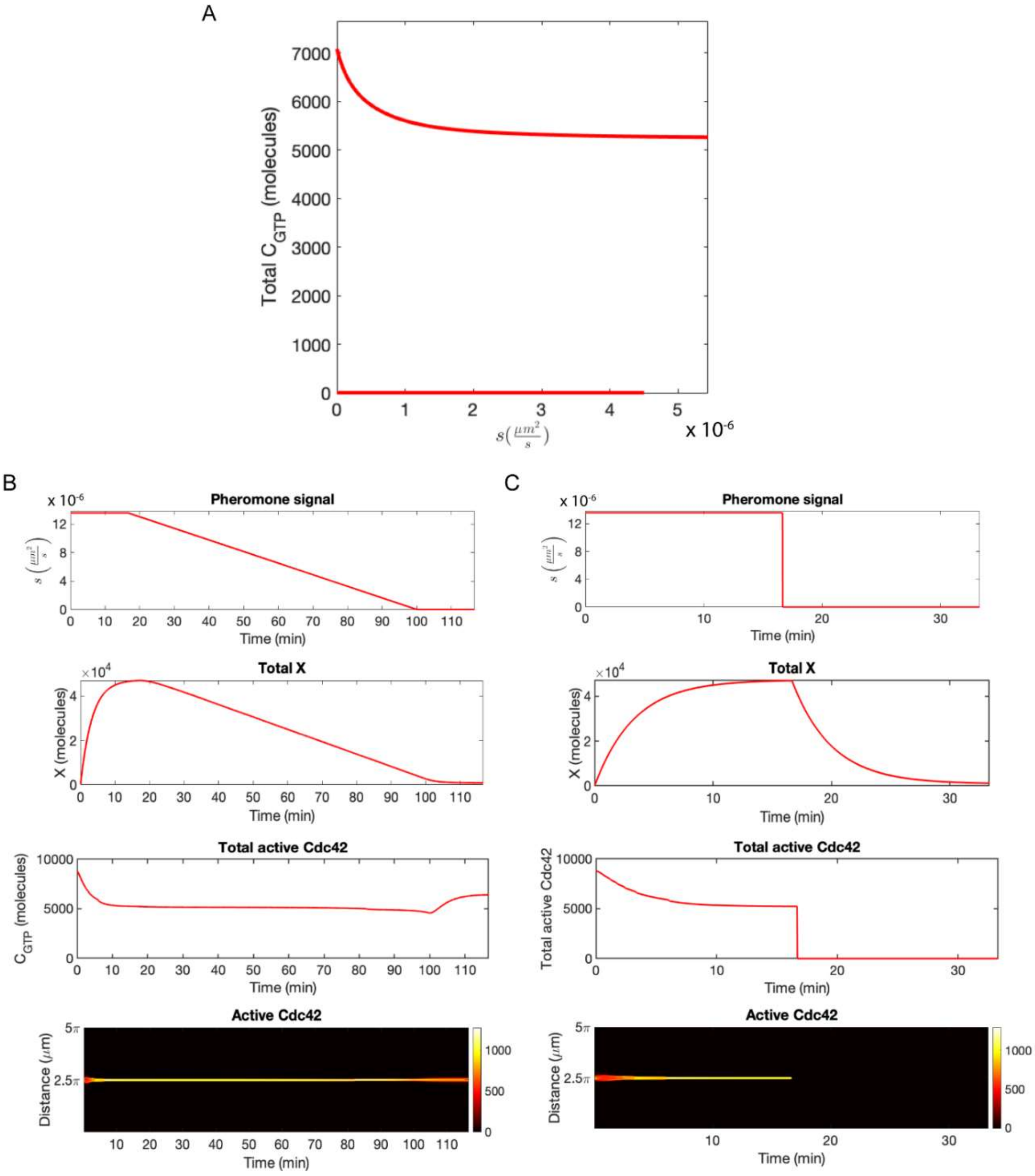

Figure S1. Results for the Incoherent feedforward model. **(A)** A single parameter bifurcation diagram in terms of pheromone signal s. **(B)** Time series for the case in which the signal s is slowly ramped down. **(C)** Same as (B), except the stimulus is removed at one step.

Movie S1. Under 5 nM pheromone, the cell established an unstable polarity patch that disassembled and reassembled dynamically.

Movie S2. The cell quickly depolarized and gradually repolarized when the pheromone concentration was decreased from 50 nM to 5 nM.

Movie S3. Spatiotemporal distribution of Cdc42-GTP after sudden reduction in the positive feedback rate λ_1. Correspond to Fig. 6C.

Movie S4. Spatiotemporal distribution of Cdc42-GTP with slow reduction in the positive feedback rate λ_1. Correspond to Fig. 6D.
